# Transcriptome profiling of chemosensory appendages in the malaria vector *Anopheles gambiae *reveals tissue- and sex-specific signatures of odor coding

**DOI:** 10.1186/1471-2164-12-271

**Published:** 2011-05-27

**Authors:** R Jason Pitts, David C Rinker, Patrick L Jones, Antonis Rokas, Laurence J Zwiebel

**Affiliations:** 1 Department of Biological Sciences, Vanderbilt University, Nashville, TN, USA; 2 Center for Human Genetics Research, Vanderbilt University, Nashville, TN, USA

## Abstract

**Background:**

Chemosensory signal transduction guides the behavior of many insects, including *Anopheles gambiae*, the major vector for human malaria in sub-Saharan Africa. To better understand the molecular basis of mosquito chemosensation we have used whole transcriptome RNA sequencing (RNA-seq) to compare transcript expression profiles between the two major chemosensory tissues, the antennae and maxillary palps, of adult female and male *An. gambiae*.

**Results:**

We compared chemosensory tissue transcriptomes to whole body transcriptomes of each sex to identify chemosensory enhanced genes. In the six data sets analyzed, we detected expression of nearly all known chemosensory genes and found them to be highly enriched in both olfactory tissues of males and females. While the maxillary palps of both sexes demonstrated strict chemosensory gene expression overlap, we observed acute differences in sensory specialization between male and female antennae. The relatively high expression levels of chemosensory genes in the female antennae reveal its role as an organ predominately assigned to chemosensation. Remarkably, the expression of these genes was highly conserved in the male antennae, but at much lower relative levels. Alternatively, consistent with a role in mating, the male antennae displayed significant enhancement of genes involved in audition, while the female enhancement of these genes was observed, but to a lesser degree.

**Conclusions:**

These findings suggest that the chemoreceptive spectrum, as defined by gene expression profiles, is largely similar in female and male *An. gambiae*. However, assuming sensory receptor expression levels are correlated with sensitivity in each case, we posit that male and female antennae are perceptive to the same stimuli, but possess inverse receptive prioritizations and sensitivities. Here we have demonstrated the use of RNA-seq to characterize the sensory specializations of an important disease vector and grounded future studies investigating chemosensory processes.

## Background

Insects rely heavily upon chemosensation, the ability to detect and react to environmental chemical cues, in virtually every aspect of their life cycle [1]. Chemosensation is critical to food source identification, predator avoidance, oviposition site selection, kin recognition, mate choice, and toxic compound avoidance. In insects, chemosensory neurons are contained within distinct tissues on many parts of the body, most conspicuously on the antennae and the maxillary palps located on the head. These appendages are decorated with sensory hairs, or sensilla, that house the neurons in which families of insect-specific receptors and other proteins transduce chemosensory signals (for reviews see [1-4]). Some insect sensory neurons have become highly specialized for the detection of single compounds, while others function more generally and are sensitive to multiple compounds [5-7]. While the physiological and cellular basis of insect chemosensation has been studied for many years, its molecular underpinnings have only recently begun to be elucidated.

In mosquitoes, host-seeking behavior is driven largely by olfaction [8,9]. *An. gambiae *females display a strong preference for human hosts (anthropophily), which contributes substantially to their ability to transmit human diseases, including malaria [8-10]. Numerous studies have shown that the antennae of *An. gambiae *are the principle chemosensory organs that respond to volatile odors [8,9]. The maxillary palps of *An. gambiae *respond to carbon dioxide, one of the major activators of mosquito upwind flight and a synergistic attractant when combined with other volatile odors [8,9,11]. The identification of chemoreceptor gene families in the *An. gambiae *genome [12,13] has facilitated the correlation of receptor expression with behavioral observations and physiological sensitivities [14-16]. Specific chemoreceptors expressed in antennal and palpal neurons of *An. gambiae *are sensitive to host odors, including volatile components produced from bacteria associated with human skin [17-19]. As a consequence, the function of select chemoreceptor genes in *An. gambiae *has been linked to semiochemicals that are integral to specific host seeking behaviors. Despite this progress, very little of the downstream signaling events and regulation of chemoreceptor function is known. Moreover, the potential chemosensory bases of sexually distinct behaviors in *An. gambiae *are poorly understood [8,20,21] and studies of male *An. gambiae *chemosensory biology are particularly lacking [20].

RNA-seq offers great potential to efficiently and comprehensively study gene expression in the chemosensory head appendages of *An. gambiae *and to provide insight into the molecular foundations of chemoreception. While several microarray-based studies have examined global transcript abundance in *An. gambiae *[22-29], none has focused exclusively on chemoreceptive tissues. Moreover, unlike microarrays and older methods, RNA-seq provides transcriptome-wide sequence coverage with unbiased, highly quantitative results [30] and greatly improved sensitivity [31,32]. To date, RNA-seq has been used to address several functional and evolutionary questions pertaining to mosquito biology [33-37].

Here we have utilized RNA-seq to quantify global abundance levels of poly-adenylated transcripts of *An. gambiae *whole adults, antennae and maxillary palps between sexes, at a life stage when females are known to host seek [8,9]. By mapping the generated short read sequences against the full set of annotated *An. gambiae *transcripts we have generated six tissue- and sex-specific transcriptome profiles (Table 1). As expected, gene families with well-established chemosensory function display antenna- or palp-enhanced expression, with antennae showing enhancement of a larger number of these genes. We also have identified numerous members of other gene families that are enhanced in either antennae or maxillary palps, such as biotransformation enzymes, transcription factors, transmembrane receptors, ion channels, transporters and proteases which are likely to function in chemosensory pathways. Our data also revealed an unanticipated level of sexual monomorphism with respect to the expression and distribution of known chemoreceptive functional classes in the antenna and the maxillary palp. Taken as a whole, this study greatly broadens our understanding of the molecular processes in peripheral sensory appendages, and establishes an agnostic, quantitative data set that can be built upon by future research.

**Table 1:** *An. gambiae *RNA-seq Mapping and Expression Data

	Overall Totals		Weighted Mapped Read Counts			Gene Expression Summary			
tissue type	reads	mapped reads (%)	transcriptome v3.6 (%)	nuclear gnm.	mito. gnm.	Gene Count	median RPKM	mean RPKM	std.dev. RPKM
female bodies	27877821	25358733 (90.96)	16606092 (59.57)	14680019	263602	12145	8.87	59.74	543.15
female antennae	25980364	24123025 (92.85)	14617276 (56.26)	15280026	80727	11722	9.38	59.22	732.65
female palps	27449612	25984839 (94.66)	15293125 (55.71)	16700334	420897	12297	10.37	56.44	496.05
male bodies	31876060	30226447 (94.82)	17603111 (55.22)	16016349	2408310	12253	8.34	54.01	424.05
male antennae	33950770	32144101 (94.68)	18231088 (53.70)	21427148	241273	11986	10.34	46.01	229.14
male palps	35705184	33339629 (93.37)	22596709 (63.29)	17625684	536952	12146	8.40	49.14	286.49

Cells in each row contain information corresponding to the **tissue type **listed. **Overall Totals: Reads**: total number of short reads generated from each sample. Mapped reads: the number (and percentage) of total reads that were mapped to the transcriptome, nuclear genome, and/or the mitochondrial genome. **Weighted Mapped Read Counts: Transcriptome v3.6**: the number (and percentage) of reads mapped to version 3.6 of the *An. gambiae *transcriptome. **Nuclear gnm.**: the number the number of reads mapped to the assembled *An. gambiae *genome. **Mito. gnm.**: the number reads mapped to the *An. gambiae *mitochondrial genome. **Gene Expression Summary: Gene count**: the total number of annotated genes in each tissue type having an **RPKM **(**R**eads **P**er **K**ilobase per **M**illion) greater than zero. **Mean, median **and **std. deviation **of the RPKM values for each tissue type.

## Methods

### Mosquito rearing

*An. gambiae sensu stricto*, which originated from Suakoko, Liberia [38], was reared as described [39]. Briefly, mosquitoes were reared in an isolated chamber at 27°C and 75% relative humidity with a photoperiod of 12:12 (L:D). Larvae were reared at low densities to ensure large adult size. Pupae were hand collected and allowed to eclose in small cages. Almost all pupae eclosed on the day after collection. The few pupae that failed to eclose were removed, such that adults in any single cage were the same age. Adult females and males were kept together in the same cages for 4-6 days and were sugar-fed with 10% sucrose *ad libitum *until the time of tissue collection. Females were not bloodfed prior to tissue collection, nor were they selected based upon any specific response to external stimuli. As a consequence of the rearing protocol, mosquitoes were intermittently exposed to the odor of their human caretakers. The vast majority of females were assumed to be mated based on numerous previous studies of cage-reared *An. gambiae *[40,41] and our own experience. Moreover, nearly all females reared as described above in our laboratory will bloodfeed when presented with an anesthetized mouse, indicating that they are physiologically competent to host seek.

### RNA isolation and sequencing

Approximately 1500 antennae or maxillary palps were hand dissected from randomly selected, age-matched cohorts of 4-6 d.o. adults (ZT10-12). Additionally, approximately 20 whole bodies of 4-6 d.o. adults were collected of each sex (ZT10-12). All collected tissues were immediately placed in RNA Later Ice (Ambion Corp.; Austin, TX) on ice prior to RNA extraction. Total RNA was isolated from each sample using RNeasy columns (Qiagen Inc.; Carlsbad, CA) according to the manufacturer's protocol. mRNA isolation and cDNA library preparation were carried out using the *Illumina *mRNA sequencing kit (*Illumina *Inc.; San Diego, CA). Libraries were sequenced using an *Illumina *Genome Analyzer II or HiSeq2000. A single biological replicate, representing a large sample size was used in the subsequent analysis.

### AgOr and AgObp reannotations

Novel *AgOrs *were identified by tBLASTn searches (http://blast.ncbi.nlm.nih.gov/Blast.cgi; default parameters) using previously identified AgOR peptides as queries. Two new candidate *AgOrs *were identified and have been named *AgOrs 80 *and *81*. Furthermore, *AgOrs 12, 67, 78 *and *79 *have been purged from the *AgOr *family as apparent duplication errors in the original assembly (Table 2). Three new candidate *AgObps *(*69, 70 *and *71*) were identified using similar tBLASTn searches and were added to the family based on two criteria: the candidate genes possessed motifs that exemplify the *Obp *family [42-45], each gene model encoded a unique transcript. Other genes resembling *Obps *were identified, but have not been included in the named members of the *AgObp *family. However we recognize the possibility that these genes may ultimately prove to be unique, or function as odor-carriers. These will be discussed in more detail below. Similarly, *AgObps 16, 17, 24, 58, 59, 60, 61*, and 65 were purged from the *AgObp *family as apparent duplication errors in assembly. All modifications to the *AgOr *and *AgObp *families have been submitted to VectorBase.

**Table 2:** Enhanced Gene Classes in *An. gambiae *Chemosensory Tissues

				enhanced >2x			
gene class	PfamA	PfamA description	# *An. gambiae*	FA	MA	FP	MP
7tm Receptor	PF00001	7tm receptor (rhodopsin family)	84	28	20	18	14
7tm Receptor	PF02949	7tm Odorant receptor (Or)	78	56	31	3	3
7tm Receptor	PF08395	7tm Chemosensory receptor (Gr)	52	1	4	3	4
7tm Receptor	PF00002	7tm receptor (Secretin family)	11	2	1	0	2
7tm Receptor	PF00003	7tm sweet-taste receptor of 3 GCPR	7	4	5	2	1
lipophilic carrier	PF01395	PBP/GOBP family	62	18	17	6	4
lipophilic carrier	PF00650	CRAL/TRIO domain	43	17	9	17	16
lipophilic carrier	PF06585	Haemolymph juvenile hormone bind. (JHBP)	24	10	5	15	9
lipophilic carrier	PF00188	Cysteine-rich secretory protein family	20	7	2	9	7
lipophilic carrier	PF03392	Insect pheromone-bind. family, A10/OS-D	7	2	2	4	1
CD36/SNMP	PF01130	CD36 family	14	5	1	7	5
channel/transporter	PF07690	Major Facilitator Superfamily	65	21	16	16	13
channel/transporter	PF00083	Sugar (and other) transporter	49	7	4	7	8
channel/transporter	PF00060	Ligand-gated ion channel	29	22	20	5	3
channel/transporter	PF00520	Ion transport protein	27	15	10	9	3
channel/transporter	PF02931	Neurotrans.-gated ion-channel ligand bind.	24	10	6	4	0
channel/transporter	PF00858	Amiloride-sensitive sodium channel	23	5	2	1	1
channel/transporter	PF01061	ABC-2 type transporter	19	10	4	12	11
channel/transporter	PF00005	ABC transporter	18	4	3	5	2
channel/transporter	PF00664	ABC transporter transmemb.	15	4	2	2	4
channel/transporter	PF07885	Ion channel	9	3	3	1	1
biotransformation	PF00067	Cytochrome P450	113	30	19	34	24
biotransformation	PF00135	Carboxylesterase	50	15	13	14	14
biotransformation	PF00043	Glutathione S-transferase, C-term.	18	6	1	4	1
biotransformation	PF02798	Glutathione S-transferase, N-term.	17	5	3	4	3
transcription factor	PF00096	Zinc finger, C2H2 type	114	21	50	21	24
transcription factor	PF00046	Homeobox domain	76	17	19	14	13
transcription factor	PF00651	BTB/POZ domain	54	17	26	5	7
transcription factor	PF00010	Helix-loop-helix DNA-binding	41	6	6	5	6
transcription factor	PF00250	Fork head domain	19	6	8	3	4
transcription factor	PF07716	Basic region leucine zipper	14	3	4	1	3
transcription factor	PF00292	\Paired box\ domain	10	3	5	3	3
transcription factor	PF00907	T-box	11	8	6	8	5
transcription factor	PF00170	bZIP transcription factor	8	3	3	2	2
transcription factor	PF00157	Pou domain - N-terminal to homeobox	4	2	3	3	1

Cells in each row contain information corresponding to the **gene class **listed. **PfamA**: PfamA family number. **PfamA description**: PfamA family description. **# in *An. gambiae***: number of genes identified in PfamA searches of *An. gambiae *transcriptome. **enhanced > 2x**: number of genes in each PfamA family that were enhanced relative to bodies in the specified tissues, relative to bodies. FA - female antennae, MA - male antennae, FP - female palps, MP - male palps

### Data processing and expression profiling

Individual *Illumina *read files were mapped to the recently updated (Dec. 2010) version of the assembled *An. gambiae *genome, to the mitochondrial genome, and to the annotated *An. gambiae *transcripts (http://www.VectorBase.org). For mapping purposes, all transcript isoforms for a given gene were condensed under that gene's AGAP designation. Prior to mapping, individual reads were quality checked and uniformly trimmed by 4 and 12 nucleotides on their 5' and 3' ends, respectively, to account for spurious adapter incorporation (5'end) and for sequencing reaction degeneration (3'-end). Mapping was carried out using seqmap software, configured to allow for a maximum of three mismatches per read. Processed mapping data was then consolidated based upon AGAP number and the results summarized by rseq software. Expression level output by rseq was reported in terms of unique reads, total weighted reads, and transcript length. Total weighted reads and AGAP transcript lengths were used to calculate a normalized transcript abundance level in units of Reads Per Kilobase per Million reads mapped (RPKMs), for every AGAP in every tissue type [32].

### PfamA categorization

Peptide sequences from AgamP3.6 conceptual peptides (n = 12669) were compared to the PfamA dataset [46], using the default e-value threshold of 1.0.

### Comparison of tissue expression profiles

Statistical significance was assigned to each pairwise tissue comparison (antennae:bodies, palps:bodies, bodies:bodies) by setting up a Fisher's Exact test, comparing the number of weighted, mapped reads for each gene to the total number of mapped reads for that tissue sample. The Agam3.6 transcript annotation contains 13319 unique, annotated transcripts and the statistical significance of the Fisher's Test was evaluated against a Bonferroni corrected p-value of 3.9 × 10^-6^.

## Results and Discussion

### RNA Sequencing and Gene Mapping

As a means of inferring gene expression in chemosensory appendages we have employed single-end short read (43bp) RNA-seq technology to characterize the relative abundances of poly-adenylated RNAs in antennae, maxillary palps and whole bodies of female and male adult mosquitoes. We established tissue-specific gene expression profiles for each of our six samples by mapping the read sequence files against the annotated *An. gambiae *transcriptome, using an approach that quantitated transcript abundance per gene and which accounted for all annotated transcripts per gene (see Materials and Methods). As our reference transcriptome, we used the AgamP3.6 version of the *An. gambiae *gene annotation, which contains 12669 protein-coding genes and 650 non-coding RNAs (http://www.VectorBase.org). For each of the tissue types assayed, we obtained an average of 30.5 million sequence reads per tissue type and mapped them to the *An. gambiae *transcriptome, nuclear and mitochondrial genomes (Table 1). Additional file 1 contains the complete RNA-seq data set described above, including the number of reads from each tissue sample that mapped to all 13319 annotated *An. gambiae *genes. On average, 57.4% of the reads per sample mapped to annotated genes, 91.5% to the nuclear genome (Table 1), and 2% to the mitochondrial genome (Table 1). Of the reads that mapped only to the genome, many of them are likely to represent unannotated 5'or 3' untranslated regions (UTRs). Moreover, there likely remain regions of the genome, most notably the Y-chromosome, where novel exons and transcripts remain [47].

**Figure 1:**
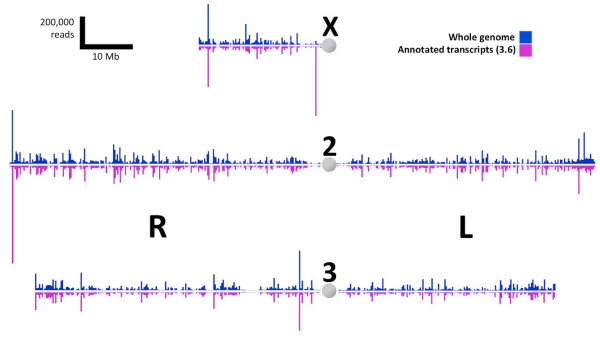
**Read coverage of *An. gambiae *genome**. Read count coverage of the nuclear genome (magenta) and of the transcriptome (blue). Vertical bars represent counts of sequence reads per 250kB interval along each of the three chromosomes.

On a whole-genome level, comparison of the mapping density of reads sequenced from the female body along all chromosomes showed a high degree of correspondence between the number of reads mapped to the nuclear genome and the number of reads mapped to the transcriptome (Figure 1). There are a few areas of asymmetry where a higher degree of mapping to either the transcriptome or to the genome was observed, most noticeably in the gene-rich autosomal telomeres and in several regions of the X chromosome (Figure 1). Greater mapping frequency to the genome represents actively transcribed regions that are unannotated as distinct genes. Greater mapping frequency to the transcriptome can generally be explained as reads that map to exon-exon junctions, which by their nature would not map to the genome. For example, the observed asymmetry in the 2R telomeric region is due to the high number of exon junction reads that mapped to two rhodopsin-family genes (Figure 1).

To quantify relative differences in gene expression levels within each tissue, we calculated a Reads Per Kilobase per Million (RPKM) reads mapped value for each gene within a sample [32]. Mean and median RPKM values for each tissue type in this study were very similar across samples, as were the number of genes showing basal or greater levels of transcription (Table 1). RPKM values spanned more than 6 orders of magnitude for each of the tissue types examined (see Additional file 1).

**Figure 2:**
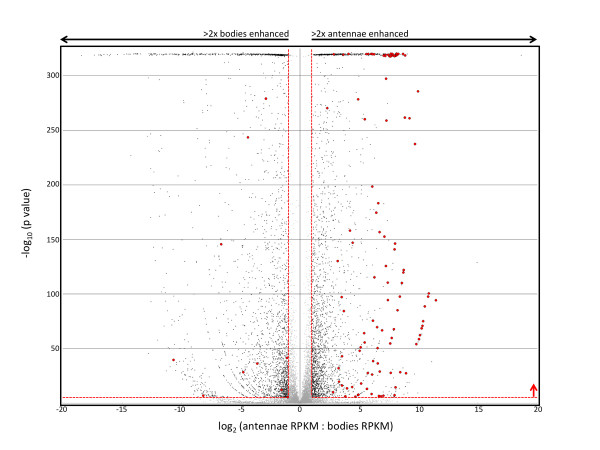
**Gene Expression in *An. gambiae *Female Antennae vs. Bodies**. Volcano plot showing the relative expression levels of genes in female whole bodies versus female antennae. The x-axis represents the log_2 _of the expression ratio (antennae RPKM: bodies RPKM) for each gene of the *An. gambiae *transcriptome. The y-axis represents the negative log_10 _of the p-value of Fisher's Exact test. Black data points (n = 2201) represent genes that were both statistically significant (red horizontal line; p< 3.9 × 10^-6^) and biologically significant (red vertical lines; greater than 2-fold difference in RPKMs). Gray data points (n = 10603) represent genes that fell outside one or both of these significance criteria. Red data points indicate the expression values of major chemosensory genes (*AgOrs, AgIrs, AgGrs*, and *AgObps*) that were statistically significant and >2-fold enhanced. RPKM values of 0.00 were transformed to 0.10 prior to calculating antennae:bodies ratios, such that those genes could also be represented on the plot.

We assessed fold-differences in transcript abundance by independently comparing ratios of RPKM values between pairs of tissues within each sex: antennae to bodies and maxillary palps to bodies. For each of these pairwise comparisons we performed a Fisher's Exact Test on counts of mapped reads and assigned statistical significance using a Bonferroni-corrected p-value (p < 3.9 × 10^-6^; see Materials and Methods). Furthermore, we have used the term "enhanced" to describe any gene that displayed at least a 2-fold, significant difference in transcript abundance between the samples being compared (Figure 2). These conservative criteria were applied to avoid false positives stemming from variations within the samples themselves, as well as to reduce the numbers of genes that were used for subsequent analyses [48,49].

### Gene Expression Profiling in Chemosensory Tissues

**Figure 3:**
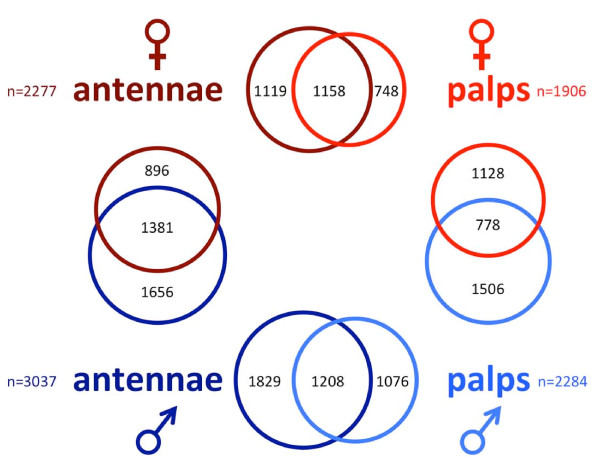
***An. gambiae *Enhanced Gene Pairwise Tissue Comparisons**. Proportional Venn diagrams showing the various pairwise comparisons made in this study. Overlap represents the subset of genes that are significantly enhanced in both tissues.

To examine global gene expression patterns, we have compared RPKM values pairwise for whole bodies versus either antennae or maxillary palps in both sexes. One such comparison is shown in Figure 2 where 4587 genes displayed enhancement in the female antenna to body comparison (Figure 2, black dots). Of those, 2277 were enhanced in the antenna (Figure 2, right half). Similarly, we found that 1906 genes were enhanced in female palps, 3037 genes were enhanced in male antennae, and 2284 genes were enhanced in male palps. These 4 gene sets formed the basis of our subsequent analyses where we compared enhanced gene profiles between chemosensory tissues and across sexes (Figure 3).

Comparing the enhanced gene sets between the female antennae and palps revealed significant overlap, with 1158 genes (61% of palp set) enhanced in both tissues (see Additional file 2). Similarly, male antennae and palps showed significant overlap with 1208 genes enhanced in both tissues (53% of palp set; see Additional file 2). Interestingly, the most well-represented gene families in both of these overlapping sets were 7-transmembrane receptors (PF00001), protein kinases (PF00069), cytochrome P450s (PF00067), trypsins (PF00089), carboxylesterases (PF00135), and potential transcription factors (PFs 00046 and 00096; see Additional files 2 and 3, bottom tables). However, we also observed several differentially enhanced gene sets between the antennae and palps (see Additional files 1 and 2). The *An. gambiae Ors *(*AgOrs*; [12]; PfamA family PF02949) were the most prevalent class in female antennae (see Additional file 2, left table) and second-most in the male antennae (see Additional file 3, left table). Other chemosensory gene families, such as ligand-gated ion channels, which include the recently identified ionotropic receptors (*AgIrs*; [13]; PF00060), and odorant binding proteins (*AgObps*; [42]; PF01395), were highly represented in the antennae (see Additional files 2 and 3). It is clear from these antennae-to-palp analyses that both extensive overlap and significant distinctions in gene expression profiles exist. The consistent identification of the same PfamA families in all enhanced gene sets implicates functional groups that can be studied in greater detail to elucidate their potential roles in mosquito chemosensation.

To evaluate gene expression patterns between sexes, we have compared female and male antennal-enhanced gene sets and palp-enhanced gene sets. In antennae, 2277 female, and 3037 male antennal-enhanced genes shared a common set of 1381 genes (Figure 3 and Additional file 4). Once again, this set included *AgOrs, AgIrs*, and *AgObps *(see Additional file 4, bottom table). Despite many commonalities in gene expression, there were also 896 female antennae-specific enhanced genes and, surprisingly, nearly 1700 male antennae-specific enhanced genes (Figure 3 and Additional file 4). In the maxillary palps, as in the antennae, considerable overlap was found in gene expression profile between the sexes. In the palp, 778 genes were common between the 1906 female palp-enhanced gene set and the 2284 male palp-enhanced gene set (Figure 3 and Additional file 5). Interestingly, the fraction of enhanced gene overlap was much lower in the palps than in the antennae; 61% of the total female antennal-enhanced set that was shared with males (see Additional file 4) while only 41% of the total female palp-enhanced set was shared with males (see Additional file 5). This result may indicate the presence of cryptic sex-specific specializations in the maxillary palps.

**Figure 4:**
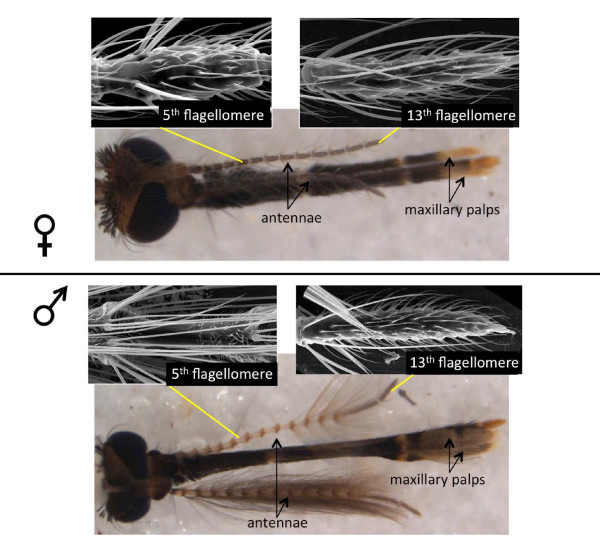
**Sexual Dimorphism in *An. gambiae *Chemosensory Tissues**. Brightfield images of *An. gambiae *female and male heads. Antennae and maxillary palps are indicated. Scanning electron micrographs show details of the fifth and thirteenth flagellomeres (segments) of female and male antennae, respectively.

Given the obvious sexual dimorphisms of *An. gambiae *antennae and maxillary palps (Figure 4) comparisons of their gene expression profiles is not necessarily straightforward. Chemosensory sensilla, and *AgOr-*containing neurons in particular, are distributed over the entire length of the female antenna, whereas male antennae house ~3-fold fewer chemosensilla that are restricted to distal segments 12 and 13 [4,50-52]. Furthermore, while female antennae are predominantly chemosensory, male antennae are also highly specialized for hearing [53,54]. Accordingly, the *An. gambiae *orthologs of the *D. melanogaster *trpV channels *Nanchung *and *inactive*, which are required for hearing in the fruit fly, were enhanced in antennae of both *An. gambiae *sexes (AGAPs 012241 and 000413, respectively; Table 2), but their expression levels were much higher in male antennae (RPKMs of 183.92 and 104.49 in males and 20.54 and 7.66 respectively, in females) [55,56]. This elevated expression of auditory-associated genes in the male antenna is consistent with male *An. gambiae *mating behavior where an acute sense of hearing facilitates the recognition of female wing beats within the context of a male swarm [40,53,54]. Given that wild females are likely to mate just once, while males swarm daily in search of a mate [20,40], the specialization shift away from olfaction and toward audition in the principle male sensory organ is reasonable, presumably as a mechanism to increase male mating success.

These comparisons also revealed multiple classes of genes beyond the known chemosensory gene families that displayed enhanced tissue expression. A detailed examination of the expression patterns of a subset of other gene families is provided in Table 2, many of which are represented in figures found in Additional files 2, 3, 4, and 5. Nearly half of the members of the large superfamily of 7-transmembrane (7tm) receptors (114 of the 241 recognized by PfamA) were enhanced in at least one of the chemosensory tissues examined (Table 2). This may indicate unrecognized roles in sensory reception or regulation of chemoreceptor neuron or accessory cell function. Importantly, efferent projections from serotonergic, or tachykinin neuroendocrine cells have been identified in mosquito chemosensory appendages [57-59]. Thus the expression of serotonin (AGAPs 002232, 002679, 004222, 004223, 007136, and 011481), and tachykinin (AGAPs 001592 and 012824) receptor homologs in *An. gambiae *antennae and maxillary palps (see Additional file 1) is consistent with a neuromodulatory role for these compounds.

Other gene families with multiple members that displayed chemosensory enhancement include the CD36 family, some members of which function in insect olfaction [60,61] and ion channels and transporters, including the recently identified chemosensory ionotropic receptors [13,62,63]. Enhanced levels of such biotransformation enzymes as carboxylesterases and cytochrome P450s could hint at a potential role in odor degradation [64-66]. Carbonic anhydrases involved in carbon dioxide detection in mammals [67] and transcription factors, including the *An. gambiae *homologs of *acj6 *and *pdm3, D. melanogaster pou*-type transcription factors involved in *DmOr *gene regulation and ORN axon targeting [68-74] were also enhanced in chemosensory tissues (Table 2).

We also identified a number of genes encoding small, soluble proteins with enhanced expression in chemosensory tissues of both sexes (Table 2). Transcripts encoding the CRAL-TRIO (PF00650), cysteine-rich secretory protein (PF00188), and haemolymph juvenile hormone binding proteins (JHBP, PF06585) were highly enhanced. To our knowledge, the first two gene families have not been linked to chemosensation, but the members of the JHBP family have been identified in screens of highly expressed genes in mosquito antennae [75,76]. Moreover the JHBP gene, *takeout*, links the circadian clock and feeding behavior in *D. melanogaster *[77], and modulates aggregation behavior in *Locusta migratoria *[78]. The extremely high expression levels of some members of these 3 gene families suggest potential chemosensory functions analogous to other soluble lipophilic carriers such as the *Obps*.

### Chemosensory Gene Families

**Figure 5:**
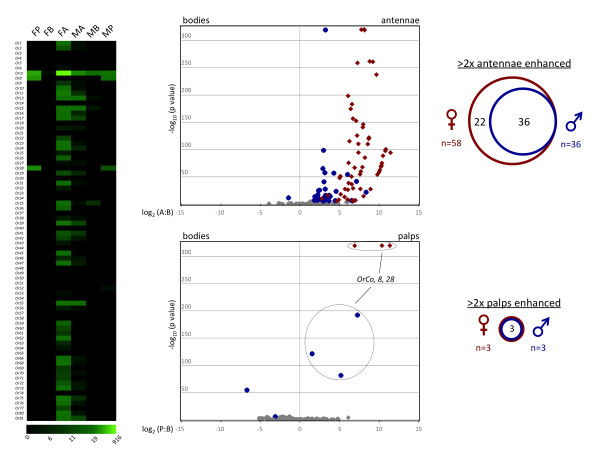
***AgOr *Expression Profile**. **Left panel **- expression profile map. Green color intensity scale (below map) indicates increasing RPKM values from left to right. (FP - female palps; FB - female bodies; FA - female antennae; MA - male antennae; MB - male bodies; MP - male palps). **Middle panels **- volcano plots showing the relative transcript expression of *AgOrs *in bodies versus antennae. Individual data points were plotted at the intersection of the log_10 _of Fisher's exact test (y-axis) and the log_2 _of the ratio of antennae (or palps) RPKM: bodies RPKM (x-axis) for each gene. Red diamonds or blue circles represent significantly enhanced *AgOrs *in antennae (top panel) or maxillary palps (bottom panel) of females and males, respectively. Gray points represent *AgOrs *that fell below the significance threshold of 3.9 × 10^-6^or the 2-fold differential expression cutoff. RPKM values of 0.00 were transformed to 0.10 prior to calculating RPKM ratios, such that those genes could also be represented on the plot. **Right panels **- Proportional Venn diagrams showing the number of *AgOrs *that are significantly enhanced in female and male antennae (top) and maxillary palps (bottom).

In light of the existing literature on the molecular mechanisms underlying the processes of peripheral chemosensation in vector mosquitoes, we examined in detail the expression patterns of *AgOrs, AgIrs, AgObps *and gustatory receptors (*AgGrs*). As expected, the vast majority of *AgOrs *were highly enhanced in antennae. Of the 76 *AgOrs*, 58 showed enhanced expression in female antennae as compared to only 36 in male antennae (Figure 5). The entire set of male-enhanced *AgOr*s was contained within the female enhanced set. None of the larval-specific *AgOrs: 37, 40, 52*, or *58*, was enhanced in adult antennae or palps, supporting previous observations [79]. In the palps, only *AgOrs8 *and *28 *and *AgOrco *(formerly *AgOr7*, recently renamed to reflect its capacity as an obligate ***Or *****co**-receptor) were enhanced in female maxillary palps (Figure 5), a result consistent with our previous study on odor coding in the *An. gambiae *maxillary palps [11]. The same 3 *AgOrs *were enhanced in male palps (Figure 5).

**Figure 6:**
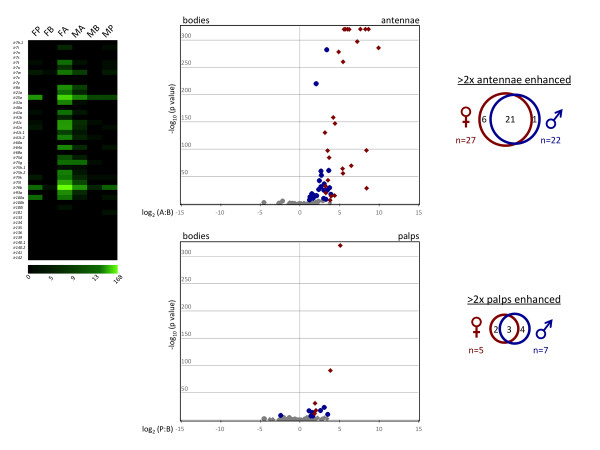
***AgIr *Expression Profile**. **Left panel **- expression profile map. Green color intensity scale (below map) indicates increasing RPKM values from left to right. Column labels same as Figure 5. **Middle panels **- volcano plots showing the relative transcript abundance of *AgIrs *in bodies versus antennae. Individual data points were plotted at the intersection of the log_10 _of Fisher's Exact test (y-axis) with the log_2 _of the ratio of antennae (or palps) RPKM: bodies RPKM (x-axis) for each gene. Red diamonds or blue circles represent significantly enhanced *AgIrs *in antennae (top panel) or maxillary palps (bottom panel) of females and males, respectively. Gray points represent *AgIrs *that fell below the significance threshold of 3.9 × 10^-6 ^or the 2-fold differential expression cutoff. RPKM values of 0.00 were transformed to 0.10 prior to calculating transcript abundance ratios, such that those genes could also be represented on the plot. **Right panels **- Proportional Venn diagrams showing the number of *AgIrs *that are significantly enhanced in female and male antennae (top) or palps (bottom).

Several members of the recently described *AgIr *gene family [13,62], displayed significant enhancement in antennae of both sexes (Figure 6), further supporting their potential roles as chemosensory receptors in *An. gambiae*. A high degree of overlap was observed between the sexes, where 21 *AgIrs *were enhanced in both. Similar to the *AgOrs*, there were many fewer *AgIrs *enhanced in the palps compared to the antennae, with 7 and 6 enhanced in female and male palps, respectively. Furthermore, the degree of overlap (3 genes) between the sexes was much less pronounced in the palp (Figure 6).

**Figure 7:**
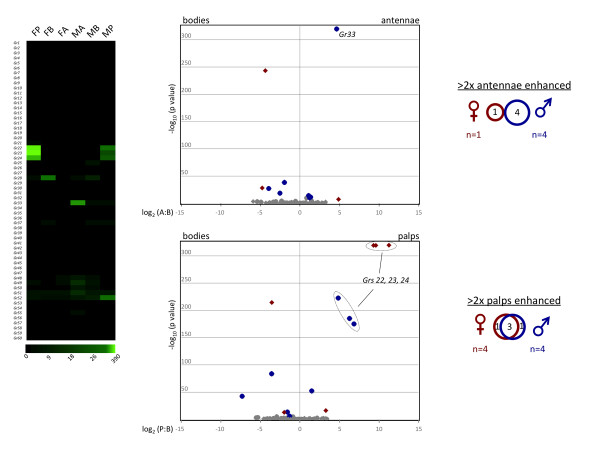
***AgGr *Expression Profile**. **Left panel **- expression profile map. Green color intensity scale (below map) indicates increasing RPKM values from left to right. Column labels same as Figure 5. **Middle panels **- volcano plots showing the relative transcript abundance of *AgGrs *in bodies versus antennae. Individual data points were plotted at the intersection of the log_10 _of Fisher's exact test (y-axis) with the log_2 _of the ratio of antennae (or palps) RPKM: bodies RPKM (x-axis) for each gene. Red diamonds or blue circles represent significantly enhanced *AgGrs *in antennae (top panel) or maxillary palps (bottom panel) of females and males, respectively. Gray points represent *AgGrs *that fell below the significance threshold of 3.9 × 10^-6 ^or the 2-fold differential expression cutoff. RPKM values of 0.00 were transformed to 0.10 prior to calculating expression ratios, such that those genes could also be represented on the plot. **Right panels **- Venn diagrams showing the number of *AgGrs *that are significantly enhanced in female and male antennae (top) or palps (bottom).

The enhanced *AgGrs *were the only class that did not overlap in the antennae between the sexes, with very few showing enhancement in either females or males (Figure 7). Only *AgGr1 *was enhanced in female antennae, while *AgGrs, 33, 48, 49*, and *50 *were enhanced in male antennae. Notably, one member of this large gene family, *AgGr33 *was strongly enhanced in male antennae (Figure 7), perhaps indicating a specialized function in males. Interestingly, *AgGr33 *shares significant homology with *D. melanogaster Gr28 *[12], some splice forms of which are expressed in non-chemosensory tissues, including the Johnston's organ [80]. In contrast to the acute sexual dimorphism displayed in the antennae, both sexes showed enhanced expression of *AgGrs 22, 23*, and *24*, in their maxillary palps (Figure 6). These three *AgGrs *are homologs of the *D. melanogaster *carbon dioxide receptors [81-83], and are expressed in capitate peg sensilla on the maxillary palps where they have been directly implicated in *An. gambiae *CO_2 _sensing [11].

**Figure 8:**
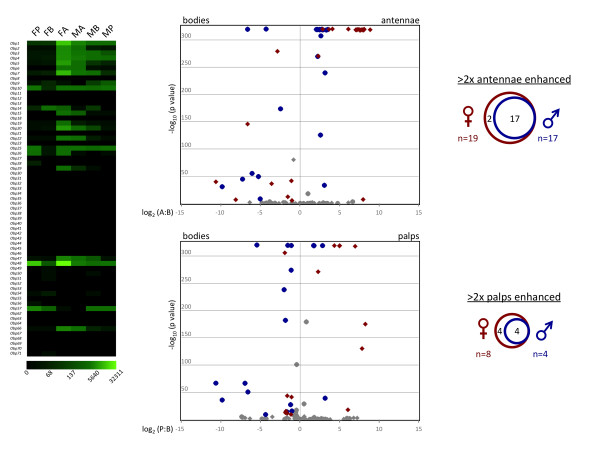
***AgObp *Expression Profile**. **Left panel **- expression profile map. Green color intensity scale (below map) indicates increasing RPKM values from left to right. Column labels same as Figure 5. **Middle panels **- volcano plots showing the relative transcript abundance of *AgObps *in bodies versus antennae. Individual data points were plotted at the intersection of the log_10 _of Fisher's exact test (y-axis) with the log_2 _of the ratio of antennae (or palps) RPKM: bodies RPKM (x-axis) for each gene. Red diamonds or blue circles represent significantly enhanced *AgObps *in antennae (top panel) or maxillary palps (bottom panel) of females and males, respectively. Gray points represent *AgObps *that fell below the significance threshold of 3.9 × 10^-6 ^or the 2-fold differential expression cutoff. RPKM values of 0.00 were transformed to 0.10 prior to calculating expression ratios, such that those genes could also be represented on the plot. **Right panels **- Venn diagrams showing the number of *AgObps *that are significantly enhanced in female and male antennae (top) or palps (bottom).

Enhanced chemosensory expression of members of the large *AgObp *family was evident across all tissues and sexes (see Additional file 1). Sixteen classical and 3 Plus-C *AgObp*s were significantly enhanced in the female antennae (Figure 8). Of these, 17 (see Additional file 1) were also significantly enhanced in the male antennae (Figure 8) including the *D. melanogaster LUSH *homolog, *AgObp4 *[84]. *AgObp19 *was the only one that demonstrated significantly enhanced expression in the female antennae and in no other tissue. In the maxillary palp, enhancement of *AgObp *transcripts also displayed substantial overlap between sexes, where the 4 male enhanced *AgObps *were all similarly elevated in females. Overall, the *AgObp *expression pattern was nearly identical between male and female chemosensory tissues (Figure 8). Several *AgObps *were also enhanced in bodies, with a greater number of them being enhanced in male bodies (Figure 8). A direct comparison of *AgObp *expression in female and male bodies revealed 17 *AgObps *that were specifically enhanced in males, while only 5 were enhanced in females. The high representation of *AgObps *in the male body without the coordinate expression of known chemoreceptors (Figures 5, 6, and 7) suggests uncharacterized roles for these *AgObps*, perhaps as general lipophilic carriers in male-specific physiology or in chemosensory processes in tarsal and wing sensilla.

In contrast, atypical *AgObp*s were not enhanced in any of the tissues examined, which is consistent with previous results suggesting that expression of this subfamily is limited to pre-adult stages [42]. With the exception of *AgObps 47, 48, 57*, which had RPKMs of >1000, expression levels of the members of the Plus-C *AgObp *subfamily was very low. Of these, it is noteworthy that *AgObp48 *was one of the most highly expressed genes (RPKM = 32311) in any tissue, with greatly elevated expression levels in both the male and female olfactory tissues (see Additional file 1). While *AgObps*, and insect *Obps *in general are among the most highly expressed gene families in chemosensory tissues their role in non-pheromone chemosensation remains largely undefined.

With regard to olfaction, it has been hypothesized that *Obps *act principally as molecular shuttles/chaperones, which deliver odorants to receptors and/or transiently protect specific odorants from biotransformation enzymes [43]. If individual *Obps *bind a subset of odorants, it is reasonable to hypothesize, that in tissues with high *Or *and therefore odor-coding complexity such as the antennae, the *Obp *landscape would need to be similarly complex in order to bind the required range of odorants. The converse would also be expected for tissues with reduced odor coding complexity such as the maxillary palp.

Female antennae showed enhanced expression of 58 conventional *AgOrs*, while only 3 *AgOrs *were enhanced in the female palp (Figure 5). Furthermore, the odorant response profiles of the palp-expressed *AgOrs 8 *and *28 *were also vastly different from the deorphanized antennal *AgOrs *[11,17,18]. These differences in *AgOr *coding capacity and their expression profiles strongly suggest that the ability of the female antennae to sense odors is much greater than the maxillary palp.

In *An. gambiae *females both the antennae and maxillary palps expressed 21 *AgObp *family members with an RPKM >10, of which 19 were found in both (see Additional file 1). While not all of these *AgObps' *transcript levels met our criteria for enhancement, they were nevertheless expressed in these tissues. Although the *AgObp *complexity was almost identical in these two appendages, they displayed vastly different *AgOr *complexity and thus odor coding capacities. This analysis confounds standing theories about *Obp *function; if a large number of *Obps *are required in the antennae for signaling, then their presence in the palp, with its more limited odor coding capacity, would appear superfluous. Given the broad expression of *AgObps *and a demonstrated lack of functional overlap between the antennae and palps, our analysis suggests that in at least some instances, *Obps *act as low-pass filters for environmental odorants rather than as specific odorant-carrier agents. Therefore, *Obps *may act to solubilize odors in some cases, but as molecular sinks in others, adding yet another dimension to peripheral odor coding. In addition, the near ubiquitous expression in both sexes of some *AgObps *suggests that they are playing completely uncharacterized roles outside of chemosensory processes.

### Diverse Roles for Chemosensory Tissues

**Figure 9:**
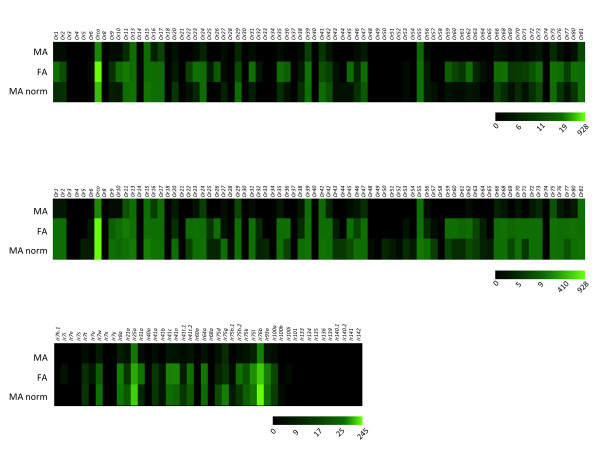
**Normalized *AgOr *and *AgIr *Expression Profiles**. **Top Panel **- Sensilla-normalized (factor 2.8) *AgOr *expression profile map. **Middle Panel **- *AgOrco*-normalized (factor 4.9) *AgOr *expression profile map. **Bottom Panel **- Sensilla-normalized (factor 4.2) *AgIr *expression profile map. Green color intensity scale (below maps) indicates increasing RPKM values from left to right. MA - male antennae RPKMs. FA - female antennae RPKMs. MA norm - normalized male antennae RPKMs.

To explore the effect of morphology on observed *AgOr *expression, we have attempted to normalize sex-specific differences in transcript abundance by scaling up male *AgOrs *in proportion to the number of female chemosensilla. *AgOrs *are expressed in the trichoid sensilla, the predominant sensillar type, and not in grooved peg (GP) sensilla [14]. Sensilla counts have indicated that female antennae house an average of 630 trichoid sensilla while male antennae house an average of 225 trichoid sensilla [4,51,85]. Multiplying the male *AgOr *RPKM levels by a factor of 2.8 (630/225), resulted in a sex-normalized, *AgOr *expression profile that was qualitatively very similar in both sexes (Figure 9, top panel), yet male *AgOr *RPKM values remained lower than those in females. Alternatively, normalization of male *AgOr *expression by a factor equal to the fold-difference in *AgOrco *RPKM levels between female and male antennae (916/186 or 4.9) was also performed. In the case of *AgOrco *normalization, *AgOr *expression profiles appeared to be very similar between sexes (Figure 9, middle panel). Based on the same logic, we also normalized *AgIr *expression in male antennae (Figure 9, bottom panel). We postulated that *AgIrs *are localized in GP sensilla neurons, as they are in *D. melanogaster *[13,61], we used a GP normalization factor of 4.2, which is the fold difference in GP numbers between female and male *An. gambiae *antennae [4]. As with *AgOrs*, the *AgIr *gene expression patterns were qualitatively similar in both sexes after normalization (Figure 9). Taken together these results suggest that male antennae express *AgOr *and *AgIr *chemoreceptor repertoires that are highly similar to those expressed in female antennae.

The *AgOr *and *AgGr *expression profiles in the maxillary palps support a similar conclusion. Although *AgOrco, AgOrs 8*, and *28*, and *AgGrs 22, 23 *and *24 *were enhanced in both sexes, their expression levels were lower in males than in females (Figures 5 and 7). As is the case for *An. gambiae *antennae, the maxillary palps are sexually dimorphic with males having about 4-fold fewer chemosensilla [4,11]. This could account for the apparent lower chemosensory gene transcript abundances in males. Normalizing male palp *AgOrs *and *AgGrs *by this factor elevated their RPKM values closer to those of females, but did not affect the qualitative observation that the identical chemoreceptors were enhanced there (data not shown). The same could be said for *AgObps *in the antennae and maxillary palps (Figures 6 and 8), which were generally more enhanced in females than in males. Assuming the expression profiles seen here are meaningful at the functional level, both sexes would potentially be receptive to a qualitatively similar odor space, with females perhaps having a lower threshold response to odors and thus greater chemoreceptive power.

There is some precedent for this hypothesis. Based on electrophysiological recordings, female *An. gambiae *antennae responded to volatiles contained in larval breeding site water at much lower thresholds than antennae of *An. gambiae *males [86]. Furthermore, at least 2 species of *Mansonia *females were attracted to mammalian hosts at greater distances than conspecific males [87]. The basis of these differences is thought to be a function of the reduced number of antennal and palpal chemosensilla in *Mansonia *males relative to conspecific females [88]. In either case, the aforementioned differences in gene expression profiles could also be functionally relevant and serve as the basis for distinguishing qualitatively and quantitatively female and male chemosensory abilities. Additionally, higher order processing could contribute significantly to sexually dimorphic behaviors, thus the perception of the same odors may elicit very different responses in the sexes. These competing hypotheses are directly testable using a combination of electrophysiological recording and behavioral response assays. Moreover, the requirement in chemoreception for any of the differentially expressed genes could potentially be explored by gene silencing.

## Conclusion

We are interested in understanding the molecular components of the chemosensory pathways that modulate the physiology and behaviors that distinguish female mosquitoes which blood-feed from males that do not. Inasmuch as differential gene expression between the sexes may serve as a potential mechanism for modulating peripheral sensitivity, we have carried out a comprehensive comparative analysis of the chemosensory transcriptomes of adult male and non-bloodfed female *An. gambiae*. Broadly, we identified several novel classes of protein coding genes whose expression is strongly biased toward chemosensory tissues, which have to date, not been associated with chemosensory pathways. Principal among these are several cytochrome P450s and a wide range of cysteine-rich secretory proteins. These genes exhibit the localization, expression and physical properties consistent with a role in semiochemical binding.

With regard to *AgObps*, a known class of semiochemical binding proteins, our data suggest that in chemosensory tissues the number of enhanced *AgObp*s is discordant with the number of enhanced chemoreceptors. This would belie the accepted and singular role for *AgObp*s in odorant binding and clearance. It is therefore reasonable to hypothesize that *AgObp*s play multi-faceted and as yet, not fully characterized, roles in the physiology of *An. gambiae*.

For the principal chemosensory gene classes, we observed an unexpected pattern of conserved *AgOr *expression between male and female antennae, lending strong empirical reinforcement to prior speculation that male and female mosquitoes share a similar range of odor coding capacity. However, the relative levels of *AgOr *transcripts were much higher in the female antennae, a finding consistent with the females' greater number of chemosensory sensilla and indicative of enhanced odor sensitivity. A similar situation was also observed in the antennal expression levels of genes associated with hearing which are expressed in both male and female antennae albeit at much higher in males. Taken together, these findings reveal the antenna of *An. gambiae *to be a bi-modal sensory appendage, one that shares a surprisingly similar suite of sensory genes between the sexes. The difference between male and female antenna seems to be less one of transcript type and more one of transcript quantity, reflective of sexually dimorphic sensory prioritizations. Male mosquito antennae are more specialized for audition and female antenna for olfaction.

We have used RNA-sequencing to conduct a high resolution and quantitative assessment of whole-transcriptome gene-expression profiles in chemosensory tissues and bodies of an organism of great medical importance. This study has begun to explore the potential of this dataset insofar as its implications for odor coding mechanisms in *An. gambiae *thereby establishing a precedent for the use of these approaches for the study of insect chemosensory processes.

## Authors' contributions

RJP, DCR and PLJ contributed equally to this work. RJP, DCR, PLJ, AR and LJZ designed the experiments. RJP, DCR and PLJ carried out the sample preparations, data analyses and wrote the manuscript with comments from AR and LJZ. All authors have read and approved the final manuscript.

## Supplementary Material

Additional file 1 ***An. gambiae *Transcriptome Expression Data**. Table of mapped reads to AgamP3.6 transcripts for all 6 data sets. **VectorBase ID**: Unique VectorBase (http://www.vectorbase.org) identification number for each *An. gambiae *gene. **transcript length**: length in base pairs of the longest annotated transcript for each gene. **chromosomal location**: chromosome arm, location of the first base pair of the initiator codon, location of the last base pair of the stop codon, reading frame (1 for plus strand or -1 for minus strand), gene name (if any). **best match to NR database (-*An. gambiae*)**: best match to non-redundant protein database (http://www.ncbi.nlm.nih.gov/BLAST/blastcgihelp.shtml#protein_databases) with *An. gambiae *proteins removed. **%ID**: percent amino acid identity between *An. gambiae *and best match peptides. **PfamA best hit**: best match to protein family identified in PfamA searches (http://pfam.janelia.org/). **e-value**: relevance value as returned in PfamA searches. **PfamA description**: protein family description. **gene**: *AgOr, AgIr, AgGr*, and *AgObp *gene families identified for easy reference. **RPKM**: normalized transcript abundance values for each gene in the indicated tissues. **unique hits**: number of RNA-seq reads that map uniquely to each gene. **total hits**: weighted number of RNA-seq reads (unique plus fraction of non-unique) that map to a given gene. RPKM values in bold type indicate significantly enhanced transcript abundance (>2-fold) in the antenna or palp relative to body for a given gene. Click here for file

Additional file 2 **Female Antennae vs. Palps Enhanced Gene Sets**. Venn diagram showing the numbers of genes that are significantly enhanced in female antennae and maxillary palps. Overlap represents the subset of genes that are significantly enhanced in both sexes. Boxes contain ranked lists of the most prevalent PfamA families in each data set. Click here for file

Additional file 3 **Male Antennae vs. Palps Enhanced Gene Sets**. Venn diagram showing the numbers of genes that are significantly enhanced in male antennae and maxillary palps. Overlap represents the subset of genes that are significantly enhanced in both sexes. Boxes contain ranked lists of the most prevalent PfamA families in each data set. Click here for file

Additional file 4 **Female vs. Male Antennae Enhanced Gene Sets**. Venn diagram showing the numbers of genes that are significantly enhanced in female and male antennae. Overlap represents the subset of genes that are significantly enhanced in both sexes. Boxes contain ranked lists of the most prevalent PfamA families in each data set. Click here for file

Additional file 5 **Female vs. Male Palps Enhanced Gene Sets**. Venn diagram showing the numbers of genes that are significantly enhanced in female and male maxillary palps. Overlap represents the subset of genes that are significantly enhanced in both sexes. Boxes contain ranked lists of the most prevalent PfamA families in each data set. Click here for file
