# Supplementary figures and images for: Transcriptome profiling of chemosensory appendages in the malaria vector Anopheles gambiae reveals tissue- and sex-specific signatures of odor coding
Source: BMC Genomics. 2011 May 27;12:271. doi: 10.1186/1471-2164-12-271 (PMC3126782; doi:10.1186/1471-2164-12-271)

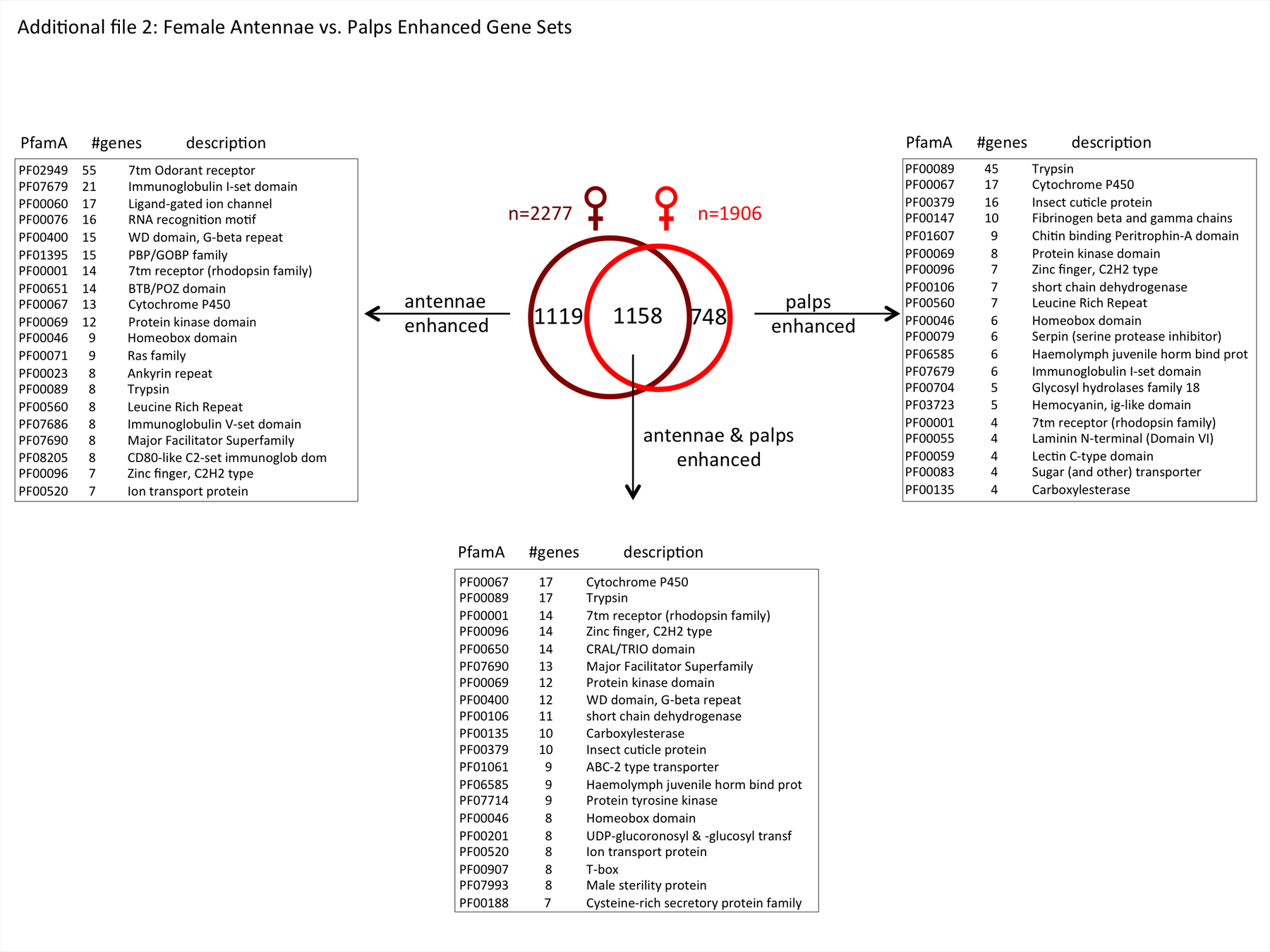

Supplement: Additional file 2 — Female Antennae vs. Palps Enhanced Gene Sets. Venn diagram showing the numbers of genes that are significantly enhanced in female antennae and maxillary palps. Overlap represents the subset of genes that are significantly enhanced in both sexes. Boxes contain ranked lists of the most prevalent PfamA families in each data set. [file 1471-2164-12-271-S2.TIFF]

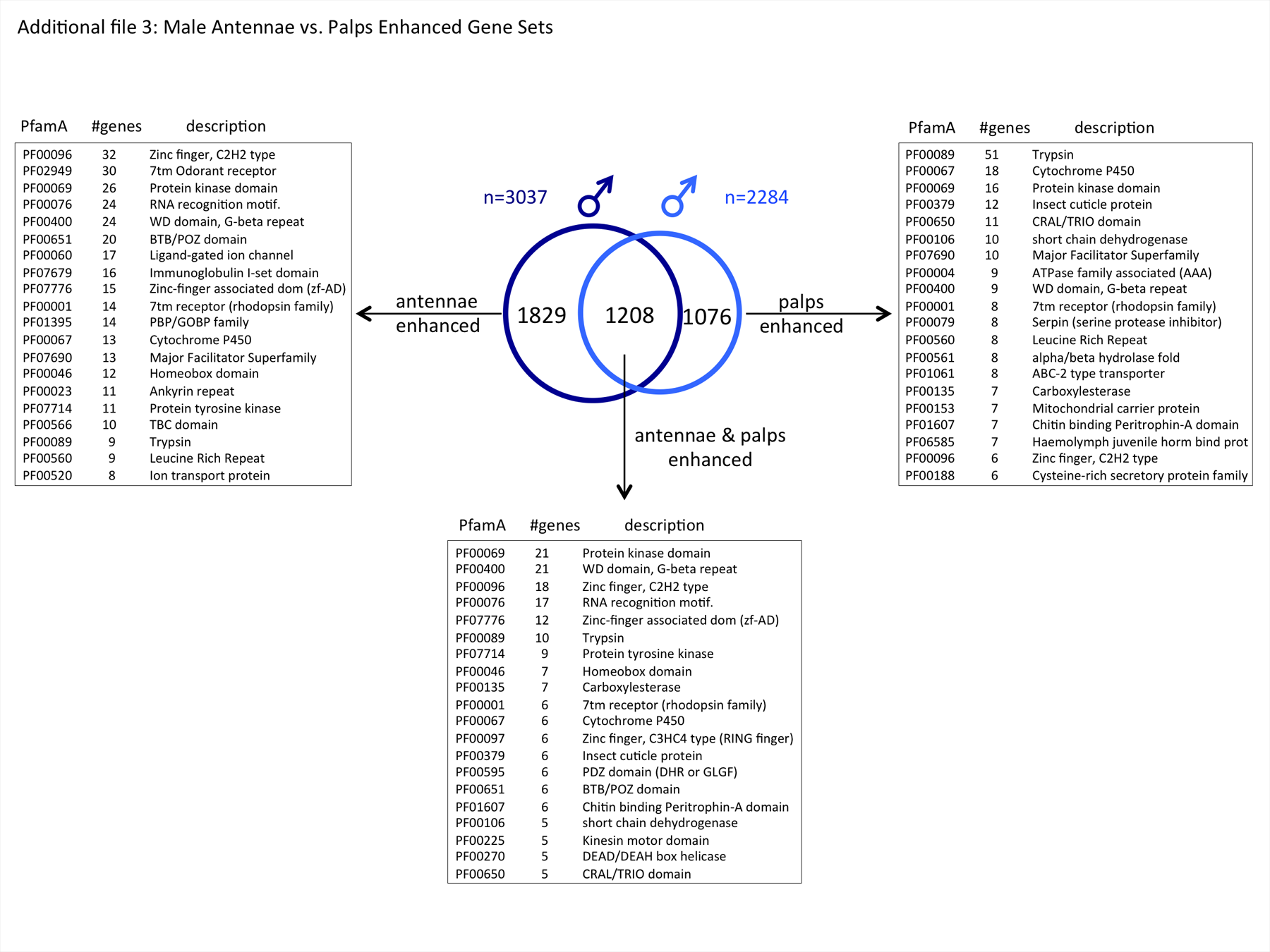

Supplement: Additional file 3 — Male Antennae vs. Palps Enhanced Gene Sets. Venn diagram showing the numbers of genes that are significantly enhanced in male antennae and maxillary palps. Overlap represents the subset of genes that are significantly enhanced in both sexes. Boxes contain ranked lists of the most prevalent PfamA families in each data set. [file 1471-2164-12-271-S3.TIFF]

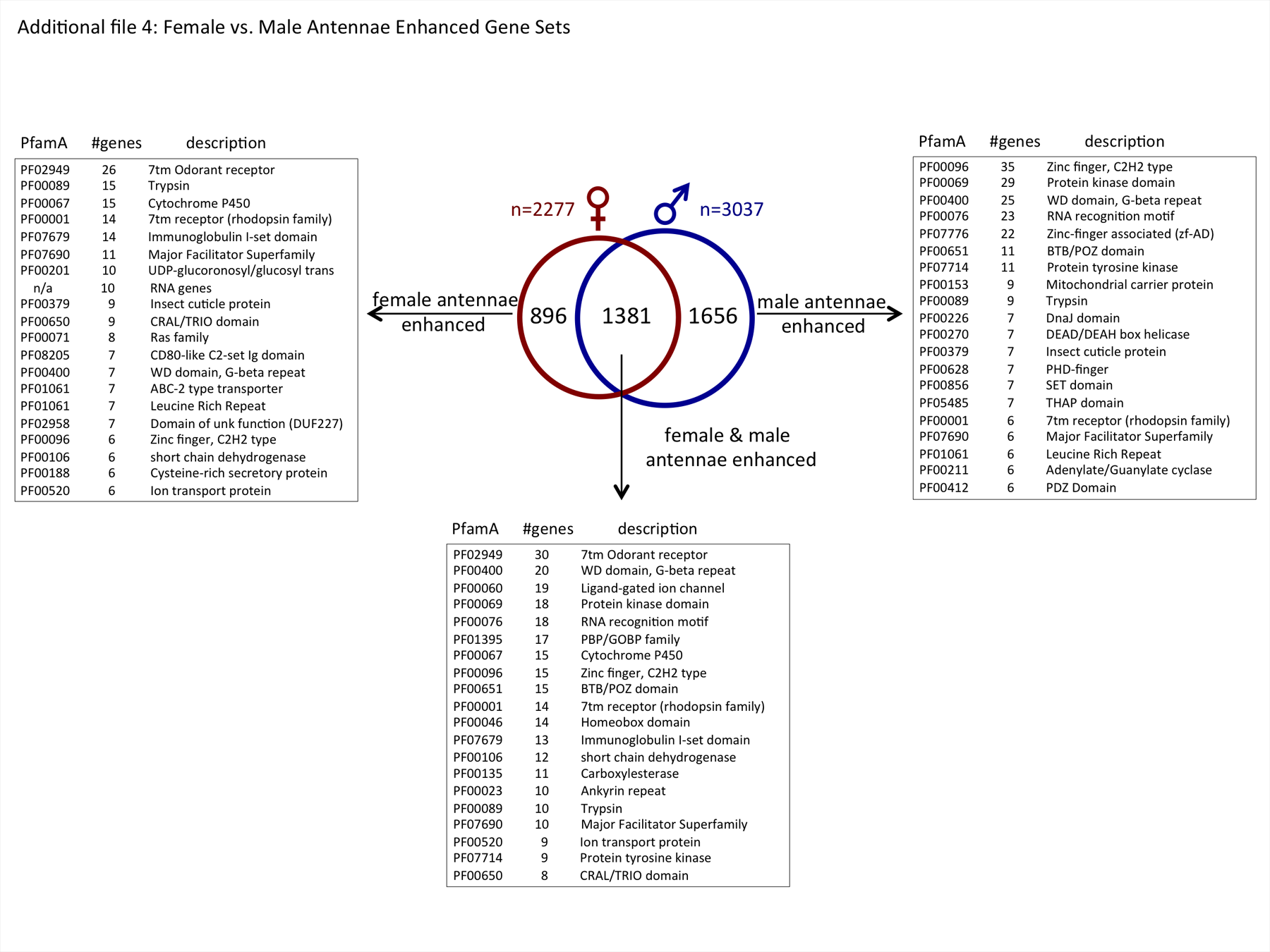

Supplement: Additional file 4 — Female vs. Male Antennae Enhanced Gene Sets. Venn diagram showing the numbers of genes that are significantly enhanced in female and male antennae. Overlap represents the subset of genes that are significantly enhanced in both sexes. Boxes contain ranked lists of the most prevalent PfamA families in each data set. [file 1471-2164-12-271-S4.TIFF]

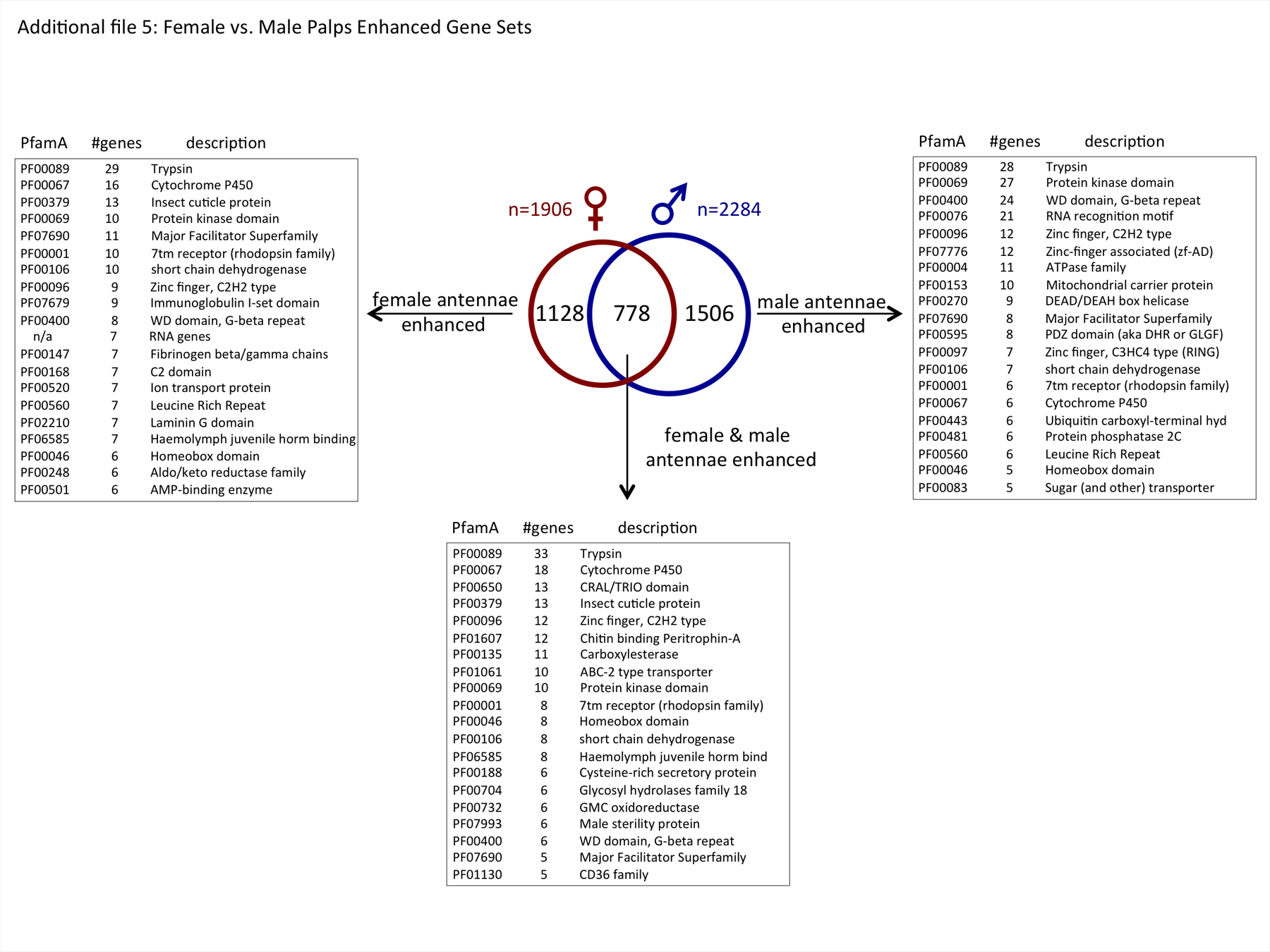

Supplement: Additional file 5 — Female vs. Male Palps Enhanced Gene Sets. Venn diagram showing the numbers of genes that are significantly enhanced in female and male maxillary palps. Overlap represents the subset of genes that are significantly enhanced in both sexes. Boxes contain ranked lists of the most prevalent PfamA families in each data set. [file 1471-2164-12-271-S5.TIFF]
